# Everybody's Page

**Published:** 1891-01-17

**Authors:** 


### Everybody's Page.

During Sheridan's last illness, the medical attendants, apprehending that they would be obliged to perform an operation on him, asked him if he had ever undergone one. "Never," replied Sheridan, "except when sitting for my picture, or having my hair cut."

The snow, against which we have all been grumbling so during the past few weeks, proved an unmixed blessing to five poor men at Strood a short time ago. When cleaning a large chimney at some cement works they were overcome by sulphur fumes, but fortunately were rescued just in time. After being rolled in the snow for a long time, in fact, being turned into veritable snow men with real eyes and limbs, they were restored eventually to consciousness.

One of the greatest, if not the greatest, of enemies which the oyster has to fear is the starfish. These voracious creatures frequently combine for the purpose of carrying on their raids, and interlace their arms so as to embrace as many shell-fish as possible. The next mode of proceedure is to protude the lower portion of the stomach through the mouth and between the valves, when the oyster is seized. If we could only plant the starfishes with a few bad oysters, and rid them of their predilection for our favourite delicacy, what benefactors we should be to the human race.

One of the finest sites in London is now being threatened by an Act which will cause it to cease for evermore to be a thing of joy. The London Tramways Company have deposited their Bill, of which official notice has been given for several years past, to extend their system across Westminster Bridge, along the Victoria Embankment, to a point nearly opposite the Charing Cross (Metropolitan District) Station. Fortunately for those who object to the numerous, and not always unsuccessful, attempts to vulgarise our streets, the construction of this extension has yet to be sanctioned.

The destruction of singing and other small birds during the cold spell with which we have been visited will have proved very large, owing to the long time during which the snow has been on the ground. Many will also, in consequence of their tameness during hard weather, have fallen victims to the vile greediness of man, as though nature had not been sufficiently hostile to them. The growing scarcity of some of our birds is a constant source of lament, and protests against the practice of eating larks seem to be as futile as they are frequent. One gentleman asserts that he would almost as soon eat little cherubim. No doubt they would prove most dainty little morsels, though our cookery book is stupidly silent on them.

Even a Chinese Emperor, it appears, is amenable to the force of public opinion, though he hardly shows his respect for it in the manner which would recommend itself to a European potentate. According to the *Pekin Gazette*, his Imperial Majesty thought fit to give explanations in reply to a remonstrance of a censor against ill-timed extravagance in erecting certain buildings for himself whilst distress prevails amongst the people in North China. Had his Majesty merely contented himself with an explanation, no doubt the censor would have gleefully flattered himself upon the success due to the possession of a virtue more exalted even than that of an Emperor. Unfortunately, however, for the censor, explanation was followed by instructions for the infliction of a severe penalty for his presumption. Even virtue has its drawbacks as well as its rewards.

What would the British or Irish farmer not give for an acre of land which would produce 974 bushels of potatos! This is the amount accredited to a farmer in Wyoming on virgin soil, rich in potash, and irrigated by water largely impregnated with saline matter.

The public owes a debt of gratitude to Mr. de Rutzen for the firm manner in which he pointed out last week to the vestries that the public safety is of higher importance than their wish not to spend extra money in clearing the roads. The munificent pay of fourpence an hour is hardly likely to attract a large number of able-bodied workmen. No doubt the staff employed by the different vestries is quite insufficient to cope with any exceptional work, but the amount of forcibly idle labour which is now available appears to be treated as a non-existent factor.

From the Shoreditch Sanitary inquiry it appears that in that district at least, if not probably in most, the hands of the sanitary staff of the vestry require strengthening in order to carry out efficiently the Infectious Diseases Notification Act and the Tenements Regulations Act. A useful result of this inquiry is to show the necessity of periodical inspections. Mere unsystematic and haphazard raids can be of no lasting use, especially in such thickly populated districts as Shoreditch, where a sufficient staff of inspectors would undoubtedly in the long run prove an economy.

The third report of the Royal Commission on Vaccination which has just been issued deals principally with the evidence of anti-vaccinators. That of Mr. Alfred Russel Wallace occupies about half the book. "Liberty," in the mind of this gentleman, "is a far greater and more important thing than science." This is a very high-sounding sentiment, but it apparently means that in the mind of Mr. Alfred Russel Wallace it is more important that a man should be free to spread a noisome disease amongst his fellow-men than that, at a slight personal discomfort to himself, science should be allowed to come to the aid of suffering humanity. We may remind Mr. Alfred Russel Wallace, that liberty and licence are not convertible terms.

A correspondent in the *Times* protests, as some people may think somewhat hardly, but with no small show of reason, against the numerous agencies now at work for the purpose of demoralising our labouring classes. There are many, and we are inclined to agree with them, who look with suspicion, amounting almost to alarm, upon the gigantic multiplication of charitable societies or schemes in this country which they conscientiously believe are steadily sapping the self-reliance and independance of Englishmen, and are rapidly adding to the numbers of that already large class of society who will not work or help themselves. There is no longer a stimulus to honest labour when you offer a man food for nothing from the soup kitchen and free education for his children; all that is necessary is to do sufficient casual work in order to purchase the necessary (?) pint of beer and ounce of tobacco. If the labourer, as is often alleged, with a few honourable exceptions, takes no pride in his work, whose fault is it? When we alleviate the temporary misfortunes of the strong and hale we are supposed to be adding to their well-being, but are we not too often only driving a fresh nail into the coffin of their thrift and self-help? To apply the bread and water of affliction in order to make the lower class of labour industrious and thrifty sounds a harsh doctrine, but this in merciful moderation is preferable to the wholesale pauperising, which seems to have such a fatal fascination for nineteenth century humanitarians.

